# *Agapostemonfasciatus* Crawford (Hymenoptera, Halictidae), a valid North American bee species ranging into southern Canada

**DOI:** 10.3897/BDJ.11.e103982

**Published:** 2023-05-19

**Authors:** Cory Sheffield

**Affiliations:** 1 Royal Saskatchewan Museum, Regina, Canada Royal Saskatchewan Museum Regina Canada

**Keywords:** sweat bees, resurrected species name, type material, DNA barcode, distribution

## Abstract

**Background:**

Sweat bees of the genus *Agapostemon* Guérin-Méneville, 1844 (Hymenoptera: Halictidae) are common and widespread in the Americas. Despite distinct morphological characters that were recognised in earlier taxonomic treatments, *Agapostemonfasciatus* Crawford, 1901 has been considered a variety of *A.melliventris* Cresson, 1874 since the 1930s and later placed into synonymy under *A.melliventris* in the early 1970s.

**New information:**

A more detailed study of morphology (including examination of type materials), distribution and genetic data (i.e. DNA barcodes) of these two taxa suggests they are not conspecific. As such, *A.fasciatus* is resurrected as a valid North American bee species. *Agapostemonfasciatus* ranges further north in North America than *A.mellivenrtis*, reaching the southern Prairies Ecozone of Canada (Alberta, Saskatchewan), while most records of *A.melliventris* are from the south-western United States and northern Mexico. More accurate distributions for both species can be modelled as specimens in collections are identified using the diagnostic features provided. However, additional work is required on the *A.melliventris* species complex in the southern United States as genetic data suggest that multiple taxa could be present.

## Introduction

Bees of the genus *Agapostemon* Guérin-Méneville, 1844 (Hymenoptera: Halictidae) are commonly encountered throughout southern Canada and all of the United States and Mexico, the Antilles and into South America (Roberts 1972, Roberts and Brooks 1987, Janjic and Packer 2003). Roberts (1972) recognised 43 species from the Americas (though see Sheffield et al. (2021)), seven of these known from Canada (Sheffield et al. 2017).

**Figure 1.**
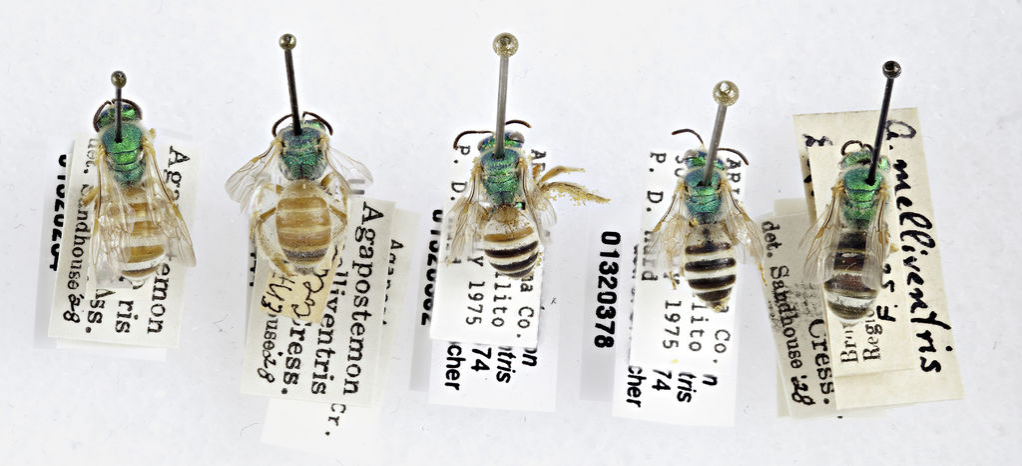
Variation in metasomal colour in female *Agapostemonmelliventris* Cresson, 1874 s. l. based on the species concept of Roberts (1972).

Sheffield et al. (2014) reported *A.melliventris* Cresson, 1874 from Saskatchewan, which was also a new record for Canada. *Agapostemonmelliventris*, a species of apparent Sonoran origin (Porter 1983), is considered rather unique amongst the genus by usually having a metasoma that is at least partially amber coloured (Fig. 1) and being one of only four species in which females have the clypeus pale maculated apically (Fig. 2a, b, c), while all other species have a clypeus that is dark apically (Fig. 2d) (see the keys of Roberts (1972)). However, the colour of the metasoma of females of *A.melliventris* s. l. varies from mostly amber to black (Cockerell 1924, Roberts 1972) (and see Fig. 1) resulting in the synonymy of two taxa under *A.melliventris*: *A.digueti* Cockerell, 1924 (synonymy by Sandhouse (1936)) and *A.fasciatus* Crawford, 1901 (synonymy by Roberts (1972)).

Females of typical *A.melliventris* have at least the first three metasomal segments almost completely amber in colour (Cresson 1874) (Fig. 3a), while the females, originally described as *A.digueti*, usually have only the first two segments amber in colour (Fig. 3b), though variation was reported even within the type material (Cockerell 1924). In addition to partially sharing the amber metasomal colouration, the females of *A.melliventris* and *A.digueti* share pale maculations on the scape (Fig. 2a, b), one of the character states assigned to this taxon (i.e. *A.melliventris* s. l.) in the morphology-based phylogeny of Janjic and Packer (2003). Females from the type series of *A.fasciatus* (Fig. 4), though sharing the clypeal colour pattern with these two taxa (Fig. 4c), differ in not having the scape maculated (Fig. 2c) and with little to no amber colouration on the metasoma (Fig. 4a, b).

The male of *A.melliventris* s. l., though known to Cockerell (1897), Titus (1900) and Hooker (1908), was not described until done so by Sandhouse (1936) and is also considered unique from most other North American *Agapostemon* in having the basal area of tergum 1 pale amber to yellow (Fig. 5a), not black (Fig. 5b) (and see Roberts (1972)). Males in the type series of *A.fasciatus* also show other distinct morphological differences from males of *A.melliventris* and *A.digueti*, including the colour of the trochanters, being metallic green in *A.fasciatus* (Fig. 6a), but entirely yellow or with slight greenish tinges on the hind leg in the other two taxa (Fig. 6b) (Sandhouse 1936).

Despite noting these morphological differences in both sexes of these taxa and commenting on differences in distribution, Sandhouse (1936) failed to recognise *A.fasciatus* (as *A.plurifasciatus* (Vachal, 1903)) as a distinct species, instead recognising it as a variety of *A.melliventris*. Subsequently, only the variation of metasomal colour of females was used by Roberts (1972) to justify his synonymy of *A.fasciatus* under *A.melliventris. Roberts (1972)* indicated a sympatric distribution of *A.fasciatus* and *A.melliventris*, though he likely did not distinguish material thoroughly, based on the distinct morphological differences that were initially described by Crawford (1901) and emphasised by Sandhouse (1936).

The purpose here is to clarify the taxonomic status of these taxa for an upcoming review of Canadian bee species by re-examining morphology and analysing COI sequences and distribution of previously recognised units of *A.melliventris* s. l. in North America.

## Materials and methods

Specimens of *A.melliventris* s. l., including type materials, from several North American collections were examined for morphological comparisons for this study, including [**CANADA**]: Royal Saskatchewan Museum [RSKM], Regina, Saskatchewan; Royal Alberta Museum [PMAE], Edmonton, Alberta; Royal British Columbia Museum [RBCM], Victoria, British Columbia; Packer Collection at York University [PCYU], Toronto, Ontario; [**UNITED STATES**]: American Museum of Natural History [AMNH], New York; United States National Museum [USNM], Washington, D.C.; University of Colorado Museum of Natural History [UCMC], Boulder, Colorado; Droege Collection, USGS Bee Inventory and Monitoring Lab [BIML], Laurel, Maryland; The University of Arizona [UAIC], Tucson, Arizona.

In addition to the materials mentioned above, records were also mined from the literature (e.g. Porter (1983)) and GBIF.org (2023)), though identifications were likely based on Roberts (1972) concept of the species.

Additional DNA barcode sequences of material from North American were obtained following previously reported methods (Sheffield et al. 2017), supplementing material already in the Barcodes of Life Data System (BOLD: Ratnasingham and Hebert (2007)) as part of an ongoing campaign to build a comprehensive DNA barcode library for the bee fauna of Canada (Sheffield et al. 2017) and the World (Packer et al. 2010). All data were mapped using SimpleMappr (Shorthouse 2010) with taxa assigned according to morphology as per Crawford (1901) and Sandhouse (1936), as described above. Only specimen data for examined specimens of *A.fasciatus* are provided for this single taxon treatment; specimens from the AMNH were identifed by Corey Smith using photos and descriptions provided by the author.

## Taxon treatments

### Agapostemon (Agapostemon) fasciatus

Crawford, 1901

314AF993-9545-53E6-81F7-25D9DAACA6C7

*Agapostemonfasciatus* Crawford, 1901: 163 [♀, ♂].
**Lectotype** ♀. Designated by J.C. Crawford in Sandhouse (1936): 79. USA, Nebraska [USNM no. 5398]. **Examined** (Fig. 4).
Halictus (Agapostemon) plurifasciatus Vachal, 1903: 101. Unnecessary replacement name.

#### Materials

- **Type status:** Other material. **Occurrence:** catalogNumber: AMNH_BEE 00039528; recordedBy: unknown; individualCount: 1; sex: male; lifeStage: adult; occurrenceID: DAC19EC9-54C0-53B5-A34C-4E0533C3C1D1; **Taxon:** scientificName: Agapostemonfasciatus; **Location:** country: United States; stateProvince: Colorado; county: Otero; locality: La Junta; decimalLatitude: 37.985; decimalLongitude: -103.54333; georeferenceProtocol: label; **Identification:** identifiedBy: C. Smith; identificationRemarks: characters for determination provided by C.S. Sheffield; **Event:** eventDate: Aug-12-1920; year: 1920; month: 8; day: 12; **Record Level:** language: en; collectionID: urn:lsid:biocol.org:col:34252; institutionCode: AMNH; collectionCode: Insects; basisOfRecord: PreservedSpecimen
- **Type status:** Other material. **Occurrence:** catalogNumber: AMNH_BEE 00214796; recordedBy: T. Cohn, P. Boone, M. A. Cazier; individualCount: 1; sex: male; lifeStage: adult; occurrenceID: 6A3B26D3-1563-525C-8613-AF30ECC7A33A; **Taxon:** scientificName: Agapostemonfasciatus; **Location:** country: United States; stateProvince: New Mexico; county: Guadalupe; locality: Newkirk; decimalLatitude: 35.06833; decimalLongitude: -104.26472; georeferenceProtocol: label; **Identification:** identifiedBy: C. Smith; identificationRemarks: characters for determination provided by C.S. Sheffield; **Event:** eventDate: Jul-15-1950; year: 1950; month: 7; day: 15; **Record Level:** language: en; collectionID: urn:lsid:biocol.org:col:34252; institutionCode: AMNH; collectionCode: Insects; basisOfRecord: PreservedSpecimen
- **Type status:** Other material. **Occurrence:** catalogNumber: AMNH_BEE 00214842; recordedBy: C. Vaurie, P. Vaurie; individualCount: 1; sex: female; lifeStage: adult; occurrenceID: B443D4BE-CBE8-5ABB-A103-3847C4F99EBC; **Taxon:** scientificName: Agapostemonfasciatus; **Location:** country: United States; stateProvince: Texas; county: Wichita; locality: 9 mi N of Electra; decimalLatitude: 34.16; decimalLongitude: -98.919; georeferenceProtocol: GPS; **Identification:** identifiedBy: C. Smith; identificationRemarks: characters for determination provided by C.S. Sheffield; **Event:** eventDate: Jun-27-1948; year: 1948; month: 6; day: 27; **Record Level:** language: en; collectionID: urn:lsid:biocol.org:col:34252; institutionCode: AMNH; collectionCode: Insects; basisOfRecord: PreservedSpecimen
- **Type status:** Other material. **Occurrence:** catalogNumber: AMNH_BEE 00214868; recordedBy: C. D. Michener; individualCount: 1; sex: female; lifeStage: adult; occurrenceID: D4F91165-1C29-5C0A-AE0E-D03DA07CABF7; **Taxon:** scientificName: Agapostemonfasciatus; **Location:** country: Mexico; stateProvince: Chihuahua; locality: 25 mi SW of Camargo; locationRemarks: D. Rockefeller Exp.; decimalLatitude: 27.4096; decimalLongitude: -105.4565; georeferenceProtocol: label; **Identification:** identifiedBy: C. Smith; identificationRemarks: characters for determination provided by C.S. Sheffield; **Event:** eventDate: Jul-14-1947; year: 1947; month: 7; day: 14; **Record Level:** language: en; collectionID: urn:lsid:biocol.org:col:34252; institutionCode: AMNH; collectionCode: Insects; basisOfRecord: PreservedSpecimen
- **Type status:** Other material. **Occurrence:** catalogNumber: AMNH_BEE 00214881; recordedBy: unknown; individualCount: 1; sex: female; lifeStage: adult; occurrenceID: 71BA74F8-58AE-57EA-AA7A-AE1E25C779A1; **Taxon:** scientificName: Agapostemonfasciatus; **Location:** country: United States; stateProvince: Texas; county: Kenedy; locality: Sarita; locationRemarks: Ex Coll. M. A. Cazier; Sand hills; decimalLatitude: 27.22338; decimalLongitude: -97.79123; **Identification:** identifiedBy: C. Smith; identificationRemarks: characters for determination provided by C.S. Sheffield; **Event:** eventDate: Dec-05-1911; year: 1911; month: 12; day: 5; **Record Level:** language: en; institutionCode: AMNH; collectionCode: Insects; basisOfRecord: PreservedSpecimen
- **Type status:** Other material. **Occurrence:** catalogNumber: AMNH_BEE 00214936; recordedBy: C. Vaurie, P. Vaurie; individualCount: 1; sex: male; lifeStage: adult; occurrenceID: FA157FA7-2652-58AB-B5E3-1078785C5DBF; **Taxon:** scientificName: Agapostemonfasciatus; **Location:** country: United States; stateProvince: Texas; county: Wichita; locality: Burkburnett, Red River; decimalLatitude: 34.0429; decimalLongitude: -98.3402; **Identification:** identifiedBy: C. Smith; identificationRemarks: characters for determination provided by C.S. Sheffield; **Event:** eventDate: Jun-26-1948; year: 1948; month: 6; day: 26; **Record Level:** language: en; institutionCode: AMNH; collectionCode: Insects; basisOfRecord: PreservedSpecimen
- **Type status:** Other material. **Occurrence:** catalogNumber: AMNH_BEE 00214954; recordedBy: C. Vaurie, P. Vaurie; individualCount: 1; sex: male; lifeStage: adult; occurrenceID: EFE76282-A890-51F5-88C2-1E4C027DDFE3; **Taxon:** scientificName: Agapostemonfasciatus; **Location:** country: United States; stateProvince: Texas; county: Wichita; locality: Burkburnett, Red River; decimalLatitude: 34.0429; decimalLongitude: -98.3402; **Identification:** identifiedBy: C. Smith; identificationRemarks: characters for determination provided by C.S. Sheffield; **Event:** eventDate: Jun-26-1948; year: 1948; month: 6; day: 26; **Record Level:** language: en; institutionCode: AMNH; collectionCode: Insects; basisOfRecord: PreservedSpecimen
- **Type status:** Other material. **Occurrence:** catalogNumber: AMNH_BEE 00214955; recordedBy: C. Vaurie, P. Vaurie; individualCount: 1; sex: male; lifeStage: adult; occurrenceID: 05C033F1-C532-5D74-B401-C4270E098556; **Taxon:** scientificName: Agapostemonfasciatus; **Location:** country: United States; stateProvince: Texas; county: Wichita; locality: Burkburnett, Red River; decimalLatitude: 34.0429; decimalLongitude: -98.3402; **Identification:** identifiedBy: C. Smith; identificationRemarks: characters for determination provided by C.S. Sheffield; **Event:** eventDate: Jun-26-1948; year: 1948; month: 6; day: 26; **Record Level:** language: en; institutionCode: AMNH; collectionCode: Insects; basisOfRecord: PreservedSpecimen
- **Type status:** Other material. **Occurrence:** catalogNumber: AMNH_BEE 00214956; recordedBy: C. Vaurie, P. Vaurie; individualCount: 1; sex: male; lifeStage: adult; occurrenceID: E697FE19-DF1E-5BA8-AD58-CA7096453D66; **Taxon:** scientificName: Agapostemonfasciatus; **Location:** country: United States; stateProvince: Texas; county: Wichita; locality: Burkburnett, Red River; decimalLatitude: 34.0429; decimalLongitude: -98.3402; **Identification:** identifiedBy: C. Smith; identificationRemarks: characters for determination provided by C.S. Sheffield; **Event:** eventDate: Jun-26-1948; year: 1948; month: 6; day: 26; **Record Level:** language: en; institutionCode: AMNH; collectionCode: Insects; basisOfRecord: PreservedSpecimen
- **Type status:** Other material. **Occurrence:** catalogNumber: AMNH_BEE 00214957; recordedBy: C. Vaurie, P. Vaurie; individualCount: 1; sex: male; lifeStage: adult; occurrenceID: 9DE14420-042C-592B-94F3-5523BEED5B00; **Taxon:** scientificName: Agapostemonfasciatus; **Location:** country: United States; stateProvince: Texas; county: Wichita; locality: Burkburnett, Red River; decimalLatitude: 34.0429; decimalLongitude: -98.3402; **Identification:** identifiedBy: C. Smith; identificationRemarks: characters for determination provided by C.S. Sheffield; **Event:** eventDate: Jun-26-1948; year: 1948; month: 6; day: 26; **Record Level:** language: en; institutionCode: AMNH; collectionCode: Insects; basisOfRecord: PreservedSpecimen
- **Type status:** Other material. **Occurrence:** catalogNumber: UCMC 0000004; recordedBy: M. Cary and/or J.C. Crawford; individualCount: 1; sex: female; lifeStage: adult; occurrenceID: AEC2CF8C-4FA3-51FC-9178-F3823312E4F3; **Taxon:** scientificName: Agapostemonfasciatus; **Location:** country: United States; stateProvince: Nebraska; locality: Lincoln; decimalLatitude: 40.808; decimalLongitude: -96.678; **Identification:** identifiedBy: C.S. Sheffield; dateIdentified: 2016; **Record Level:** basisOfRecord: PreservedSpecimen
- **Type status:** Other material. **Occurrence:** catalogNumber: Type 5398 / USNM ENT 00536742; recordedBy: M. Cary; individualCount: 1; sex: female; lifeStage: adult; occurrenceID: 431F3783-1230-5389-9A9E-17B8938C7A09; **Taxon:** scientificName: Agapostemonfasciatus; **Location:** country: United States; stateProvince: Nebraska; locality: Lincoln; decimalLatitude: 40.808; decimalLongitude: -96.678; **Identification:** identifiedBy: J.C. Crawford; dateIdentified: 1901; **Event:** year: 1900; month: 9; day: 30; **Record Level:** basisOfRecord: PreservedSpecimen
- **Type status:** Other material. **Occurrence:** catalogNumber: Paratype No.5398 / USNM; recordedBy: M. Cary and/or J.C. Crawford; individualCount: 1; sex: female; lifeStage: adult; occurrenceID: E80A82FD-5CBD-5522-A28C-B263EDEDED83; **Taxon:** scientificName: Agapostemonfasciatus; **Location:** country: United States; stateProvince: Nebraska; locality: Lincoln; decimalLatitude: 40.808; decimalLongitude: -96.678; **Identification:** identifiedBy: C.S. Sheffield; dateIdentified: 2016; **Event:** year: 1900; month: 5; **Record Level:** basisOfRecord: PreservedSpecimen
- **Type status:** Other material. **Occurrence:** catalogNumber: Paratype No.5398 / USNM; recordedBy: J.C. Crawford; individualCount: 1; sex: male; lifeStage: adult; occurrenceID: E5B8D491-1E73-5BB8-92BC-EB8BAF6EB401; **Taxon:** scientificName: Agapostemonfasciatus; **Location:** country: United States; stateProvince: Nebraska; locality: Lincoln; decimalLatitude: 40.808; decimalLongitude: -96.678; **Identification:** identifiedBy: C.S. Sheffield; dateIdentified: 2016; **Event:** year: 1900; month: 9; day: 30; **Record Level:** basisOfRecord: PreservedSpecimen
- **Type status:** Other material. **Occurrence:** catalogNumber: Paratype No.5398 / USNM; recordedBy: J.C. Crawford; individualCount: 1; sex: male; lifeStage: adult; occurrenceID: 1F43CF59-62ED-5E1E-87BE-C71B2E77A2D8; **Taxon:** scientificName: Agapostemonfasciatus; **Location:** country: United States; stateProvince: Nebraska; locality: Lincoln; decimalLatitude: 40.808; decimalLongitude: -96.678; **Identification:** identifiedBy: C.S. Sheffield; dateIdentified: 2016; **Event:** year: 1900; month: 9; day: 30; **Record Level:** basisOfRecord: PreservedSpecimen
- **Type status:** Other material. **Occurrence:** catalogNumber: Paratype No.5398 / USNM; recordedBy: J.C. Crawford; individualCount: 1; sex: male; lifeStage: adult; occurrenceID: 3EBF0CE7-9ED1-55A0-8C55-4706680EC575; **Taxon:** scientificName: Agapostemonfasciatus; **Location:** country: United States; stateProvince: Nebraska; locality: Lincoln; decimalLatitude: 40.808; decimalLongitude: -96.678; **Identification:** identifiedBy: C.S. Sheffield; dateIdentified: 2016; **Event:** year: 1900; month: 9; day: 30; **Record Level:** basisOfRecord: PreservedSpecimen
- **Type status:** Other material. **Occurrence:** catalogNumber: Paratype No.5398 / USNM; recordedBy: J.C. Crawford; individualCount: 1; sex: male; lifeStage: adult; occurrenceID: 71B533D1-6060-58AF-95FA-B801F320E978; **Taxon:** scientificName: Agapostemonfasciatus; **Location:** country: United States; stateProvince: Nebraska; locality: Lincoln; decimalLatitude: 40.808; decimalLongitude: -96.678; **Identification:** identifiedBy: C.S. Sheffield; dateIdentified: 2016; **Event:** year: 1900; month: 9; **Record Level:** basisOfRecord: PreservedSpecimen
- **Type status:** Other material. **Occurrence:** catalogNumber: Paratype No.5398 / USNM; recordedBy: J.C. Crawford; individualCount: 1; sex: male; lifeStage: adult; occurrenceID: 7BDBC9C2-C8EB-5863-81C1-61D7E025FFF4; **Taxon:** scientificName: Agapostemonfasciatus; **Location:** country: United States; stateProvince: Nebraska; locality: Lincoln; decimalLatitude: 40.808; decimalLongitude: -96.678; **Identification:** identifiedBy: C.S. Sheffield; dateIdentified: 2016; **Event:** year: 1900; month: 9; day: 30; **Record Level:** basisOfRecord: PreservedSpecimen
- **Type status:** Other material. **Occurrence:** catalogNumber: USGS-DRO 240369; recordedBy: S. Droege; individualCount: 1; sex: female; lifeStage: adult; occurrenceID: A651D576-93B5-5B3A-B0BA-A1F7E1BE661D; **Taxon:** scientificName: Agapostemonfasciatus; **Location:** country: United States; stateProvince: South Dakota; county: Shannon Co.; decimalLatitude: 43.5221; decimalLongitude: -102.4489; georeferenceProtocol: label; **Identification:** identifiedBy: C.S. Sheffield; dateIdentified: 2016; **Event:** year: 2011; month: 7; day: 10; **Record Level:** basisOfRecord: PreservedSpecimen
- **Type status:** Other material. **Occurrence:** catalogNumber: USGS-DRO 243568; recordedBy: S. Droege; individualCount: 1; sex: female; lifeStage: adult; occurrenceID: EAFA4A99-9D88-5ACD-96A4-0C26F8ABCDEB; **Taxon:** scientificName: Agapostemonfasciatus; **Location:** country: United States; stateProvince: South Dakota; county: Shannon Co.; decimalLatitude: 43.5265; decimalLongitude: -102.4431; georeferenceProtocol: label; **Identification:** identifiedBy: C.S. Sheffield; dateIdentified: 2016; **Event:** year: 2011; month: 7; day: 10; **Record Level:** basisOfRecord: PreservedSpecimen
- **Type status:** Other material. **Occurrence:** catalogNumber: USGS-DRO 240457; recordedBy: S. Droege; individualCount: 1; sex: female; lifeStage: adult; occurrenceID: 2AB1970F-392B-5ABA-A329-D50F2F5A7D73; **Taxon:** scientificName: Agapostemonfasciatus; **Location:** country: United States; stateProvince: South Dakota; county: Shannon Co.; decimalLatitude: 43.5221; decimalLongitude: -102.4489; georeferenceProtocol: label; **Identification:** identifiedBy: C.S. Sheffield; dateIdentified: 2016; **Event:** year: 2011; month: 7; day: 10; **Record Level:** basisOfRecord: PreservedSpecimen
- **Type status:** Other material. **Occurrence:** catalogNumber: USGS-DRO 240453; recordedBy: S. Droege; individualCount: 1; sex: female; lifeStage: adult; occurrenceID: 0F27E857-EADF-5D2D-B0E0-172AEAFD6B82; **Taxon:** scientificName: Agapostemonfasciatus; **Location:** country: United States; stateProvince: South Dakota; county: Shannon Co.; decimalLatitude: 43.5221; decimalLongitude: -102.4489; georeferenceProtocol: label; **Identification:** identifiedBy: C.S. Sheffield; dateIdentified: 2016; **Event:** year: 2011; month: 7; day: 10; **Record Level:** basisOfRecord: PreservedSpecimen
- **Type status:** Other material. **Occurrence:** catalogNumber: USGS-DRO 225099; recordedBy: B. Crew; individualCount: 1; sex: female; lifeStage: adult; occurrenceID: A2BAA54A-DD06-53EB-BFF6-45F30648742C; **Taxon:** scientificName: Agapostemonfasciatus; **Location:** country: United States; stateProvince: South Dakota; county: Pennington Co.; decimalLatitude: 43.7865; decimalLongitude: -102.088; georeferenceProtocol: label; **Identification:** identifiedBy: C.S. Sheffield; dateIdentified: 2016; **Event:** year: 2011; month: 6; day: 28; **Record Level:** basisOfRecord: PreservedSpecimen
- **Type status:** Other material. **Occurrence:** catalogNumber: USGS-DRO 243578; recordedBy: S. Droege; individualCount: 1; sex: female; lifeStage: adult; occurrenceID: C016DD96-08F1-5240-BF15-90B7C279F2EB; **Taxon:** scientificName: Agapostemonfasciatus; **Location:** country: United States; stateProvince: South Dakota; county: Shannon Co.; decimalLatitude: 43.5265; decimalLongitude: -102.4431; georeferenceProtocol: label; **Identification:** identifiedBy: C.S. Sheffield; dateIdentified: 2016; **Event:** year: 2011; month: 7; day: 10; **Record Level:** basisOfRecord: PreservedSpecimen
- **Type status:** Other material. **Occurrence:** catalogNumber: USGS-DRO 243588; recordedBy: S. Droege; individualCount: 1; sex: female; lifeStage: adult; occurrenceID: 0C0D8132-8FA6-5DAA-9580-6C8C134B79A5; **Taxon:** scientificName: Agapostemonfasciatus; **Location:** country: United States; stateProvince: South Dakota; county: Shannon Co.; decimalLatitude: 43.5265; decimalLongitude: -102.4431; georeferenceProtocol: label; **Identification:** identifiedBy: C.S. Sheffield; dateIdentified: 2016; **Event:** year: 2011; month: 7; day: 10; **Record Level:** basisOfRecord: PreservedSpecimen
- **Type status:** Other material. **Occurrence:** catalogNumber: USGS-DRO 249374; recordedBy: B. Crew; individualCount: 1; sex: female; lifeStage: adult; occurrenceID: 069D7978-92BA-5ADE-855D-0483D9EA912C; **Taxon:** scientificName: Agapostemonfasciatus; **Location:** country: United States; stateProvince: South Dakota; county: Shannon Co.; decimalLatitude: 43.6482; decimalLongitude: -102.7239; georeferenceProtocol: label; **Identification:** identifiedBy: C.S. Sheffield; dateIdentified: 2016; **Event:** year: 2011; month: 6; day: 4; **Record Level:** basisOfRecord: PreservedSpecimen
- **Type status:** Other material. **Occurrence:** catalogNumber: USGS-DRO 243541; recordedBy: S. Droege; individualCount: 1; sex: female; lifeStage: adult; occurrenceID: 6A386B70-929E-577F-A0E6-4CBF10EAD01F; **Taxon:** scientificName: Agapostemonfasciatus; **Location:** country: United States; stateProvince: South Dakota; county: Shannon Co.; decimalLatitude: 43.5265; decimalLongitude: -102.4431; georeferenceProtocol: label; **Identification:** identifiedBy: C.S. Sheffield; dateIdentified: 2016; **Event:** year: 2011; month: 7; day: 10; **Record Level:** basisOfRecord: PreservedSpecimen
- **Type status:** Other material. **Occurrence:** catalogNumber: USGS-DRO 243550; recordedBy: S. Droege; individualCount: 1; sex: female; lifeStage: adult; occurrenceID: DAFAD72E-3EFF-56CC-8628-3A4A2071B091; **Taxon:** scientificName: Agapostemonfasciatus; **Location:** country: United States; stateProvince: South Dakota; county: Shannon Co.; decimalLatitude: 43.5265; decimalLongitude: -102.4431; georeferenceProtocol: label; **Identification:** identifiedBy: C.S. Sheffield; dateIdentified: 2016; **Event:** year: 2011; month: 7; day: 10; **Record Level:** basisOfRecord: PreservedSpecimen
- **Type status:** Other material. **Occurrence:** catalogNumber: USGS-DRO 249426; recordedBy: B. Crew; individualCount: 1; sex: female; lifeStage: adult; occurrenceID: 94856752-52C6-5620-B0B7-E82C3A303D81; **Taxon:** scientificName: Agapostemonfasciatus; **Location:** country: United States; stateProvince: South Dakota; county: Shannon Co.; decimalLatitude: 43.6482; decimalLongitude: -102.7239; georeferenceProtocol: label; **Identification:** identifiedBy: C.S. Sheffield; dateIdentified: 2016; **Event:** year: 2011; month: 6; day: 4; **Record Level:** basisOfRecord: PreservedSpecimen
- **Type status:** Other material. **Occurrence:** catalogNumber: USGS-DRO 243540; recordedBy: S. Droege; individualCount: 1; sex: female; lifeStage: adult; occurrenceID: 8057A88E-F4F7-5874-86DE-5EA7956D6845; **Taxon:** scientificName: Agapostemonfasciatus; **Location:** country: United States; stateProvince: South Dakota; county: Shannon Co.; decimalLatitude: 43.5265; decimalLongitude: -102.4431; georeferenceProtocol: label; **Identification:** identifiedBy: C.S. Sheffield; dateIdentified: 2016; **Event:** year: 2011; month: 7; day: 10; **Record Level:** basisOfRecord: PreservedSpecimen
- **Type status:** Other material. **Occurrence:** catalogNumber: USGS-DRO 244089; recordedBy: B. Crew; individualCount: 1; sex: female; lifeStage: adult; occurrenceID: 7DBB9407-D519-5BDF-9444-1D1C861F2A4D; **Taxon:** scientificName: Agapostemonfasciatus; **Location:** country: United States; stateProvince: South Dakota; county: Pennington Co.; decimalLatitude: 43.7165; decimalLongitude: -102.0387; georeferenceProtocol: label; **Identification:** identifiedBy: C.S. Sheffield; dateIdentified: 2016; **Event:** year: 2011; month: 6; day: 3; **Record Level:** basisOfRecord: PreservedSpecimen
- **Type status:** Other material. **Occurrence:** catalogNumber: USGS-DRO 243634; recordedBy: S. Droege; individualCount: 1; sex: female; lifeStage: adult; occurrenceID: D0188499-7ACD-5CCC-B772-C004E9F68AD5; **Taxon:** scientificName: Agapostemonfasciatus; **Location:** country: United States; stateProvince: South Dakota; county: Shannon Co.; decimalLatitude: 43.5265; decimalLongitude: -102.4431; georeferenceProtocol: label; **Identification:** identifiedBy: C.S. Sheffield; dateIdentified: 2016; **Event:** year: 2011; month: 7; day: 10; **Record Level:** basisOfRecord: PreservedSpecimen
- **Type status:** Other material. **Occurrence:** catalogNumber: UCMC 0264261; recordedBy: U.N. Lanham; individualCount: 1; sex: female; lifeStage: adult; occurrenceID: C2EF84D9-EAB0-5EB9-9AA6-F471653A46E9; **Taxon:** scientificName: Agapostemonfasciatus; **Location:** country: United States; stateProvince: Wyoming; locality: Upton; decimalLatitude: 44.1; decimalLongitude: -104.627; **Identification:** identifiedBy: C.S. Sheffield; dateIdentified: 2016; **Event:** year: 1988; month: 6; day: 17; **Record Level:** basisOfRecord: PreservedSpecimen
- **Type status:** Other material. **Occurrence:** catalogNumber: UCMC 0264262; recordedBy: U.N. Lanham; individualCount: 1; sex: female; lifeStage: adult; occurrenceID: 259DE543-25B8-5DFD-8CB0-A335B7ED3CF6; **Taxon:** scientificName: Agapostemonfasciatus; **Location:** country: United States; stateProvince: Wyoming; locality: Upton; decimalLatitude: 44.1; decimalLongitude: -104.627; **Identification:** identifiedBy: C.S. Sheffield; dateIdentified: 2016; **Event:** year: 1988; month: 6; day: 17; **Record Level:** basisOfRecord: PreservedSpecimen
- **Type status:** Other material. **Occurrence:** catalogNumber: UCMC 0264263; recordedBy: U.N. Lanham; individualCount: 1; sex: female; lifeStage: adult; occurrenceID: 70F651F9-8615-5095-A9B7-01F692770E8C; **Taxon:** scientificName: Agapostemonfasciatus; **Location:** country: United States; stateProvince: Wyoming; locality: 21 mi S. Newcastle; decimalLatitude: 43.545; decimalLongitude: -104.196; **Identification:** identifiedBy: C.S. Sheffield; dateIdentified: 2016; **Event:** year: 1988; month: 6; day: 18; **Record Level:** basisOfRecord: PreservedSpecimen
- **Type status:** Other material. **Occurrence:** catalogNumber: UCMC 0264264; recordedBy: P. Robinson; individualCount: 1; sex: female; lifeStage: adult; occurrenceID: 745C43C4-9212-59BD-96FB-805D2EC1564F; **Taxon:** scientificName: Agapostemonfasciatus; **Location:** country: United States; stateProvince: Wyoming; county: Johnson Co.; locality: nr. Sussex, 3 mi S of Dry Fork mouth; decimalLatitude: 43.687; decimalLongitude: -106.276; **Identification:** identifiedBy: C.S. Sheffield; dateIdentified: 2016; **Event:** year: 1991; month: 7; day: 3; **Record Level:** basisOfRecord: PreservedSpecimen
- **Type status:** Other material. **Occurrence:** catalogNumber: UCMC 0264265; recordedBy: P. Robinson; individualCount: 1; sex: female; lifeStage: adult; occurrenceID: 13F31842-5C01-5504-B399-CF8ABBD3B1BE; **Taxon:** scientificName: Agapostemonfasciatus; **Location:** country: United States; stateProvince: Wyoming; county: Johnson Co.; locality: nr. Sussex, 3 mi S of Dry Fork mouth; decimalLatitude: 43.687; decimalLongitude: -106.276; **Identification:** identifiedBy: C.S. Sheffield; dateIdentified: 2016; **Event:** year: 1991; month: 7; day: 3; **Record Level:** basisOfRecord: PreservedSpecimen
- **Type status:** Other material. **Occurrence:** catalogNumber: UCMC 0264266; recordedBy: P. Robinson; individualCount: 1; sex: female; lifeStage: adult; occurrenceID: A6355899-12B3-532A-8DEB-3C89D0855CB4; **Taxon:** scientificName: Agapostemonfasciatus; **Location:** country: United States; stateProvince: Wyoming; county: Johnson Co.; locality: nr. Sussex, 3 mi S of Dry Fork mouth; decimalLatitude: 43.687; decimalLongitude: -106.276; **Identification:** identifiedBy: C.S. Sheffield; dateIdentified: 2016; **Event:** year: 1991; month: 7; day: 3; **Record Level:** basisOfRecord: PreservedSpecimen
- **Type status:** Other material. **Occurrence:** catalogNumber: UCMC 0264267; recordedBy: P. Robinson; individualCount: 1; sex: female; lifeStage: adult; occurrenceID: 640DC5C9-64FA-5548-A56D-8CC468233059; **Taxon:** scientificName: Agapostemonfasciatus; **Location:** country: United States; stateProvince: Wyoming; county: Johnson Co.; locality: nr. Sussex, 3 mi S of Dry Fork mouth; decimalLatitude: 43.687; decimalLongitude: -106.276; **Identification:** identifiedBy: C.S. Sheffield; dateIdentified: 2016; **Event:** year: 1991; month: 7; day: 3; **Record Level:** basisOfRecord: PreservedSpecimen
- **Type status:** Other material. **Occurrence:** catalogNumber: UCMC 0264268; recordedBy: P. Robinson; individualCount: 1; sex: female; lifeStage: adult; occurrenceID: F6C7FC0A-DB20-533E-A2C9-2F9D4484B17A; **Taxon:** scientificName: Agapostemonfasciatus; **Location:** country: United States; stateProvince: Wyoming; county: Johnson Co.; locality: nr. Sussex, 3 mi S of Dry Fork mouth; decimalLatitude: 43.687; decimalLongitude: -106.276; **Identification:** identifiedBy: C.S. Sheffield; dateIdentified: 2016; **Event:** year: 1991; month: 7; day: 3; **Record Level:** basisOfRecord: PreservedSpecimen
- **Type status:** Other material. **Occurrence:** catalogNumber: UCMC 0264269; recordedBy: P. Robinson; individualCount: 1; sex: female; lifeStage: adult; occurrenceID: 5C0D6317-D9A6-5014-8B12-81380FFC4566; **Taxon:** scientificName: Agapostemonfasciatus; **Location:** country: United States; stateProvince: Wyoming; county: Johnson Co.; locality: nr. Sussex, 3 mi S of Dry Fork mouth; decimalLatitude: 43.687; decimalLongitude: -106.276; **Identification:** identifiedBy: C.S. Sheffield; dateIdentified: 2016; **Event:** year: 1991; month: 7; day: 3; **Record Level:** basisOfRecord: PreservedSpecimen
- **Type status:** Other material. **Occurrence:** catalogNumber: UCMC 0264270; recordedBy: P. Robinson; individualCount: 1; sex: female; lifeStage: adult; occurrenceID: CE534890-C565-557E-B6BE-BAE8010A5952; **Taxon:** scientificName: Agapostemonfasciatus; **Location:** country: United States; stateProvince: Wyoming; county: Johnson Co.; locality: nr. Sussex, 3 mi S of Dry Fork mouth; decimalLatitude: 43.687; decimalLongitude: -106.276; **Identification:** identifiedBy: C.S. Sheffield; dateIdentified: 2016; **Event:** year: 1991; month: 7; day: 3; **Record Level:** basisOfRecord: PreservedSpecimen
- **Type status:** Other material. **Occurrence:** catalogNumber: UCMC 0264271; recordedBy: P. Robinson; individualCount: 1; sex: female; lifeStage: adult; occurrenceID: 0F9FFF9D-94E3-5F31-82C4-FA4DA6EE788E; **Taxon:** scientificName: Agapostemonfasciatus; **Location:** country: United States; stateProvince: Wyoming; county: Johnson Co.; locality: nr. Sussex, 3 mi S of Dry Fork mouth; decimalLatitude: 43.687; decimalLongitude: -106.276; **Identification:** identifiedBy: C.S. Sheffield; dateIdentified: 2016; **Event:** year: 1991; month: 7; day: 3; **Record Level:** basisOfRecord: PreservedSpecimen
- **Type status:** Other material. **Occurrence:** catalogNumber: UCMC 0264272; recordedBy: U.N. Lanham; individualCount: 1; sex: female; lifeStage: adult; occurrenceID: FEEA7278-385B-54D0-BDA3-57D81CBE9CF6; **Taxon:** scientificName: Agapostemonfasciatus; **Location:** country: United States; stateProvince: Wyoming; locality: Upton; decimalLatitude: 44.1; decimalLongitude: -104.627; **Identification:** identifiedBy: C.S. Sheffield; dateIdentified: 2016; **Event:** year: 1988; month: 6; day: 17; **Record Level:** basisOfRecord: PreservedSpecimen
- **Type status:** Other material. **Occurrence:** catalogNumber: UCMC 0264273; recordedBy: S. Vogel; individualCount: 1; sex: female; lifeStage: adult; occurrenceID: A758ABDA-CE7C-5E16-B8B9-1C2367D9FC55; **Taxon:** scientificName: Agapostemonfasciatus; **Location:** country: United States; stateProvince: Montana; county: Carbon Co.; locality: Cottonwood Crk. 8 mi S. Bridger; decimalLatitude: 45.2; decimalLongitude: -108.86; **Identification:** identifiedBy: C.S. Sheffield; dateIdentified: 2016; **Event:** year: 1980; month: 8; day: 30; **Record Level:** basisOfRecord: PreservedSpecimen
- **Type status:** Other material. **Occurrence:** catalogNumber: UCMC 0264274; recordedBy: P. Robinson; individualCount: 1; sex: female; lifeStage: adult; occurrenceID: 11ADB813-57E6-57EE-AB91-B400010AD63C; **Taxon:** scientificName: Agapostemonfasciatus; **Location:** country: United States; stateProvince: Wyoming; county: Natrona Co.; locality: Badwater Camp; decimalLatitude: 43.32; decimalLongitude: -107.42; **Identification:** identifiedBy: C.S. Sheffield; dateIdentified: 2016; **Event:** year: 1979; month: 7; day: 27; **Record Level:** basisOfRecord: PreservedSpecimen
- **Type status:** Other material. **Occurrence:** catalogNumber: UCMC 0264275; recordedBy: U.N. Lanham; individualCount: 1; sex: female; lifeStage: adult; occurrenceID: B177A951-0120-5FFB-B837-1C27A65FC8D8; **Taxon:** scientificName: Agapostemonfasciatus; **Location:** country: United States; stateProvince: Wyoming; county: Johnson Co.; locality: 21 mi SE Kaycee; decimalLatitude: 43.46; decimalLongitude: -106.398; **Identification:** identifiedBy: C.S. Sheffield; dateIdentified: 2016; **Event:** year: 1981; month: 7; day: 1; **Record Level:** basisOfRecord: PreservedSpecimen
- **Type status:** Other material. **Occurrence:** catalogNumber: UCMC 0264276; recordedBy: H.E. Evans; individualCount: 1; sex: female; lifeStage: adult; occurrenceID: 5FE83B0D-A680-5F4D-B60F-8C72C6E2F386; **Taxon:** scientificName: Agapostemonfasciatus; **Location:** country: United States; stateProvince: Colorado; county: Bent Co.; locality: Hasty; decimalLatitude: 38.11; decimalLongitude: -102.957; **Identification:** identifiedBy: C.S. Sheffield; dateIdentified: 2016; **Event:** year: 1974; month: 6; day: 26; **Record Level:** basisOfRecord: PreservedSpecimen
- **Type status:** Other material. **Occurrence:** catalogNumber: UCMC 0264277; recordedBy: U. Lanham; individualCount: 1; sex: female; lifeStage: adult; occurrenceID: 33239BF4-318C-5E34-BDCD-340AFDA29898; **Taxon:** scientificName: Agapostemonfasciatus; **Location:** country: United States; stateProvince: Colorado; county: Bent Co.; locality: 2 mi S Hasty; decimalLatitude: 38.08; decimalLongitude: -102.958; **Identification:** identifiedBy: C.S. Sheffield; dateIdentified: 2016; **Event:** year: 1975; month: 6; day: 26; **Record Level:** basisOfRecord: PreservedSpecimen
- **Type status:** Other material. **Occurrence:** catalogNumber: UCMC 0264278; recordedBy: U.N. Lanham; individualCount: 1; sex: female; lifeStage: adult; occurrenceID: 9C5C2A2C-E1B3-53DE-88EE-EC32A4B85BCC; **Taxon:** scientificName: Agapostemonfasciatus; **Location:** country: United States; stateProvince: Colorado; county: Bent Co.; locality: 2 mi S Hasty; decimalLatitude: 38.08; decimalLongitude: -102.958; **Identification:** identifiedBy: C.S. Sheffield; dateIdentified: 2016; **Event:** year: 1975; month: 6; day: 26; **Record Level:** basisOfRecord: PreservedSpecimen
- **Type status:** Other material. **Occurrence:** catalogNumber: UCMC 0264279; recordedBy: U.N. Lanham; individualCount: 1; sex: female; lifeStage: adult; occurrenceID: D7D82E2E-2DFE-51DA-986E-335177985C4B; **Taxon:** scientificName: Agapostemonfasciatus; **Location:** country: United States; stateProvince: Colorado; county: Bent Co.; locality: 2 mi S Hasty; decimalLatitude: 38.08; decimalLongitude: -102.958; **Identification:** identifiedBy: C.S. Sheffield; dateIdentified: 2016; **Event:** year: 1975; month: 6; day: 25; **Record Level:** basisOfRecord: PreservedSpecimen
- **Type status:** Other material. **Occurrence:** catalogNumber: UCMC 0264280; recordedBy: M.A. Carriker, Jr.; individualCount: 1; sex: female; lifeStage: adult; occurrenceID: 543B8AAF-33C5-53FC-8EA4-2C4C2ABADB10; **Taxon:** scientificName: Agapostemonfasciatus; **Location:** country: United States; stateProvince: Nebraska; locality: Lincoln; decimalLatitude: 40.808; decimalLongitude: -96.678; **Identification:** identifiedBy: C.S. Sheffield; dateIdentified: 2016; **Event:** year: 1901; month: 8; day: 4; **Record Level:** basisOfRecord: PreservedSpecimen
- **Type status:** Other material. **Occurrence:** catalogNumber: UCMC 0264281; recordedBy: M.A. Carriker, Jr.; individualCount: 1; sex: male; lifeStage: adult; occurrenceID: 22905317-ECB3-520F-AF0B-21F574DC7537; **Taxon:** scientificName: Agapostemonfasciatus; **Location:** country: United States; stateProvince: Nebraska; locality: Lincoln; decimalLatitude: 40.808; decimalLongitude: -96.678; **Identification:** identifiedBy: C.S. Sheffield; dateIdentified: 2016; **Event:** year: 1901; month: 8; day: 4; **Record Level:** basisOfRecord: PreservedSpecimen
- **Type status:** Other material. **Occurrence:** catalogNumber: UCMC 0264282; recordedBy: H.G. Rodeck, M.T. James; individualCount: 1; sex: male; lifeStage: adult; occurrenceID: 7840D7E4-7232-5448-9CD5-81AE0F7FFB6D; **Taxon:** scientificName: Agapostemonfasciatus; **Location:** country: United States; stateProvince: Colorado; locality: N of Lolita; decimalLatitude: 38.28; decimalLongitude: -103.59; **Identification:** identifiedBy: C.S. Sheffield; dateIdentified: 2016; **Event:** year: 1933; month: 8; day: 8; **Record Level:** basisOfRecord: PreservedSpecimen
- **Type status:** Other material. **Occurrence:** catalogNumber: UCMC 0264283; recordedBy: H.G. Rodeck; individualCount: 1; sex: male; lifeStage: adult; occurrenceID: 2C3D6360-8E52-586F-93AA-1693356A99A7; **Taxon:** scientificName: Agapostemonfasciatus; **Location:** country: United States; stateProvince: Colorado; county: Boulder Co.; locality: West of Niwot; decimalLatitude: 40.104; decimalLongitude: -105.17; **Identification:** identifiedBy: C.S. Sheffield; dateIdentified: 2016; **Event:** year: 1946; month: 9; day: 13; **Record Level:** basisOfRecord: PreservedSpecimen
- **Type status:** Other material. **Occurrence:** catalogNumber: UCMC 0264284; recordedBy: H.G. Rodeck; individualCount: 1; sex: male; lifeStage: adult; occurrenceID: DBB6A87D-EB5A-5F11-B27A-A0CFF164B6F0; **Taxon:** scientificName: Agapostemonfasciatus; **Location:** country: United States; stateProvince: Colorado; county: Boulder Co.; locality: West of Niwot; decimalLatitude: 40.104; decimalLongitude: -105.17; **Identification:** identifiedBy: C.S. Sheffield; dateIdentified: 2016; **Event:** year: 1946; month: 9; day: 13; **Record Level:** basisOfRecord: PreservedSpecimen
- **Type status:** Other material. **Occurrence:** catalogNumber: UCMC 0264285; recordedBy: H.G. Rodeck; individualCount: 1; sex: male; lifeStage: adult; occurrenceID: D5867E02-BFF0-5D9B-A1CA-368958AC6B3F; **Taxon:** scientificName: Agapostemonfasciatus; **Location:** country: United States; stateProvince: Colorado; county: Crowley Co.; locality: 3 mi NE Lolita; decimalLatitude: 38.28; decimalLongitude: -103.59; **Identification:** identifiedBy: C.S. Sheffield; dateIdentified: 2016; **Event:** year: 1957; month: 8; day: 7; **Record Level:** basisOfRecord: PreservedSpecimen
- **Type status:** Other material. **Occurrence:** catalogNumber: UCMC 0264286; recordedBy: H.G. Rodeck; individualCount: 1; sex: male; lifeStage: adult; occurrenceID: 07DD260A-C4C3-5978-8577-DF818EACB193; **Taxon:** scientificName: Agapostemonfasciatus; **Location:** country: United States; stateProvince: Colorado; county: Crowley Co.; locality: 3 mi NE Lolita; decimalLatitude: 38.28; decimalLongitude: -103.59; **Identification:** identifiedBy: C.S. Sheffield; dateIdentified: 2016; **Event:** year: 1957; month: 8; day: 7; **Record Level:** basisOfRecord: PreservedSpecimen
- **Type status:** Other material. **Occurrence:** catalogNumber: UCMC 0264287; recordedBy: U.N. Lanham; individualCount: 1; sex: male; lifeStage: adult; occurrenceID: BED286AB-E213-588B-AF21-20DF161E29B5; **Taxon:** scientificName: Agapostemonfasciatus; **Location:** country: United States; stateProvince: Colorado; county: Bent Co.; locality: 2 mi S Hasty; decimalLatitude: 38.08; decimalLongitude: -102.958; **Identification:** identifiedBy: C.S. Sheffield; dateIdentified: 2016; **Event:** year: 1974; month: 7; day: 19; **Record Level:** basisOfRecord: PreservedSpecimen
- **Type status:** Other material. **Occurrence:** catalogNumber: UCMC 0264288; recordedBy: U.N. Lanham; individualCount: 1; sex: male; lifeStage: adult; occurrenceID: BDECBC25-9FCE-5674-B5BD-8847AEA9B9FE; **Taxon:** scientificName: Agapostemonfasciatus; **Location:** country: United States; stateProvince: Colorado; county: Bent Co.; locality: 2 mi S Hasty; decimalLatitude: 38.08; decimalLongitude: -102.958; **Identification:** identifiedBy: C.S. Sheffield; dateIdentified: 2016; **Event:** year: 1974; month: 8; day: 13; **Record Level:** basisOfRecord: PreservedSpecimen
- **Type status:** Other material. **Occurrence:** catalogNumber: UCMC 0264289; recordedBy: S. Vogel; individualCount: 1; sex: male; lifeStage: adult; occurrenceID: EDF28B65-A205-5776-B9BF-A656C65A88BA; **Taxon:** scientificName: Agapostemonfasciatus; **Location:** country: United States; stateProvince: Montana; county: Carbon Co.; locality: Cottonwood Crk. 8 mi S. Bridger; decimalLatitude: 45.2; decimalLongitude: -108.86; **Identification:** identifiedBy: C.S. Sheffield; dateIdentified: 2016; **Event:** year: 1980; month: 8; day: 30; **Record Level:** basisOfRecord: PreservedSpecimen
- **Type status:** Other material. **Occurrence:** catalogNumber: UCMC 0264290; recordedBy: V.A. Scott; individualCount: 1; sex: male; lifeStage: adult; occurrenceID: 42E679AE-98FC-5D54-8909-C4108FA3471A; **Taxon:** scientificName: Agapostemonfasciatus; **Location:** country: United States; stateProvince: Colorado; county: Crowley Co.; locality: 2 mi SW Sugar City, Lake Meredith; decimalLatitude: 38.2122; decimalLongitude: -103.691; **Identification:** identifiedBy: C.S. Sheffield; dateIdentified: 2016; **Event:** year: 2002; month: 8; day: 20; **Record Level:** basisOfRecord: PreservedSpecimen
- **Type status:** Other material. **Occurrence:** catalogNumber: UCMC 0264291; recordedBy: V.A. Scott; individualCount: 1; sex: male; lifeStage: adult; occurrenceID: 3BDD669B-7CDC-5875-A9BB-1A741307C26B; **Taxon:** scientificName: Agapostemonfasciatus; **Location:** country: United States; stateProvince: Colorado; county: Crowley Co.; locality: 2 mi SW Sugar City, Lake Meredith; decimalLatitude: 38.2122; decimalLongitude: -103.691; **Identification:** identifiedBy: C.S. Sheffield; dateIdentified: 2016; **Event:** year: 2002; month: 8; day: 20; **Record Level:** basisOfRecord: PreservedSpecimen
- **Type status:** Other material. **Occurrence:** catalogNumber: RSKM_ENT_E-90141; recordedBy: D. Larson; individualCount: 1; sex: female; lifeStage: adult; occurrenceID: 5000133E-E8BE-5042-88BB-BEA3634EE9E6; **Taxon:** scientificName: Agapostemonfasciatus; **Location:** country: Canada; stateProvince: Saskatchewan; locality: Grasslands NP, West Block; decimalLatitude: 49.2; decimalLongitude: -107.7; georeferenceProtocol: label; **Identification:** identifiedBy: C.S. Sheffield; dateIdentified: 2016; **Event:** year: 2009; month: 6; day: 13; **Record Level:** basisOfRecord: PreservedSpecimen
- **Type status:** Other material. **Occurrence:** catalogNumber: PMAE00125959; recordedBy: M. Buck; individualCount: 1; sex: female; lifeStage: adult; occurrenceID: EA1E75C7-B1B8-53B0-BFA6-21B7DC1C70E7; **Taxon:** scientificName: Agapostemonfasciatus; **Location:** country: Canada; stateProvince: Alberta; county: Cypress Co.; locality: Onefour, HRNA; decimalLatitude: 49.01; decimalLongitude: -110.44611; georeferenceProtocol: label; **Identification:** identifiedBy: C.S. Sheffield; dateIdentified: 2016; **Event:** year: 2013; month: 7; day: 15; **Record Level:** basisOfRecord: PreservedSpecimen
- **Type status:** Other material. **Occurrence:** catalogNumber: PMAE00125960; recordedBy: M. Buck; individualCount: 1; sex: female; lifeStage: adult; occurrenceID: 93F63DAD-1240-57CD-BA71-F1D9880FED57; **Taxon:** scientificName: Agapostemonfasciatus; **Location:** country: Canada; stateProvince: Alberta; county: Cypress Co.; locality: Onefour, HRNA; decimalLatitude: 49.01; decimalLongitude: -110.44611; georeferenceProtocol: label; **Identification:** identifiedBy: C.S. Sheffield; dateIdentified: 2016; **Event:** year: 2013; month: 7; day: 15; **Record Level:** basisOfRecord: PreservedSpecimen
- **Type status:** Other material. **Occurrence:** catalogNumber: PMAE00125961; recordedBy: M. Buck; individualCount: 1; sex: female; lifeStage: adult; occurrenceID: A5DE7AD1-ABA5-5F1F-A780-68E99D365784; **Taxon:** scientificName: Agapostemonfasciatus; **Location:** country: Canada; stateProvince: Alberta; county: Cypress Co.; locality: Onefour, HRNA; decimalLatitude: 49.01; decimalLongitude: -110.44611; georeferenceProtocol: label; **Identification:** identifiedBy: C.S. Sheffield; dateIdentified: 2016; **Event:** year: 2013; month: 7; day: 15; **Record Level:** basisOfRecord: PreservedSpecimen
- **Type status:** Other material. **Occurrence:** catalogNumber: PMAE00125962; recordedBy: M. Buck; individualCount: 1; sex: female; lifeStage: adult; occurrenceID: 06463A2A-FDA1-5748-A692-6426C6C97DF1; **Taxon:** scientificName: Agapostemonfasciatus; **Location:** country: Canada; stateProvince: Alberta; county: Cypress Co.; locality: Onefour, HRNA; decimalLatitude: 49.01; decimalLongitude: -110.44611; georeferenceProtocol: label; **Identification:** identifiedBy: C.S. Sheffield; dateIdentified: 2016; **Event:** year: 2013; month: 7; day: 15; **Record Level:** basisOfRecord: PreservedSpecimen
- **Type status:** Other material. **Occurrence:** catalogNumber: PMAE00125963; recordedBy: M. Buck; individualCount: 1; sex: female; lifeStage: adult; occurrenceID: 1E7EEEF7-9BFB-5247-B453-CFBC39A4B13B; **Taxon:** scientificName: Agapostemonfasciatus; **Location:** country: Canada; stateProvince: Alberta; county: Cypress Co.; locality: Onefour, HRNA; decimalLatitude: 49.01; decimalLongitude: -110.44611; georeferenceProtocol: label; **Identification:** identifiedBy: C.S. Sheffield; dateIdentified: 2016; **Event:** year: 2013; month: 7; day: 15; **Record Level:** basisOfRecord: PreservedSpecimen
- **Type status:** Other material. **Occurrence:** catalogNumber: PMAE00125964; recordedBy: M. Buck; individualCount: 1; sex: female; lifeStage: adult; occurrenceID: F40E5018-1936-5500-B53B-A574370A2F27; **Taxon:** scientificName: Agapostemonfasciatus; **Location:** country: Canada; stateProvince: Alberta; county: Cypress Co.; locality: Onefour, HRNA; decimalLatitude: 49.01; decimalLongitude: -110.44611; georeferenceProtocol: label; **Identification:** identifiedBy: C.S. Sheffield; dateIdentified: 2016; **Event:** year: 2013; month: 7; day: 15; **Record Level:** basisOfRecord: PreservedSpecimen

#### Diagnosis

The female of *A.fasciatus* can be distinguished from all other North American (i.e. north of Mexico) members of the subgenusAgapostemon (*sensu* Janjic and Packer (2003)), except *A.melliventris* and *A.peninsularis* by the pale apical maculation on the clypeus (Fig. 2); within the subgenusNotagapostemon, *A.nasutus* and *A.leunculus* also share this feature (Roberts 1972, Janjic and Packer 2003). It can be separated from *A.melliventris* by lacking the pale maculation on the scape (Fig. 2c), which is present in *A.melliventris* (Fig. 2a, b) and the entirely dark metasoma (Fig. 4a, b), which is partly to mostly amber coloured in *A.melliventris* (Fig. 3).

Males of *A.fasciatus* can be separated from all other North American *Agapostemon*, except *A.melliventris* in having the basal area of tergum 1 coloured pale amber to yellow (Fig. 5a), not black (Fig. 5b). Males of *A.fasciatus* differ from *A.melliventris* by the colour of the trochanters, being metallic green in *A.fasciatus* (Fig. 6a), but entirely non-metallic yellow in *A.melliventris* (Fig. 6b). Differences in genitalia are not apparent.

**Figure 7.**
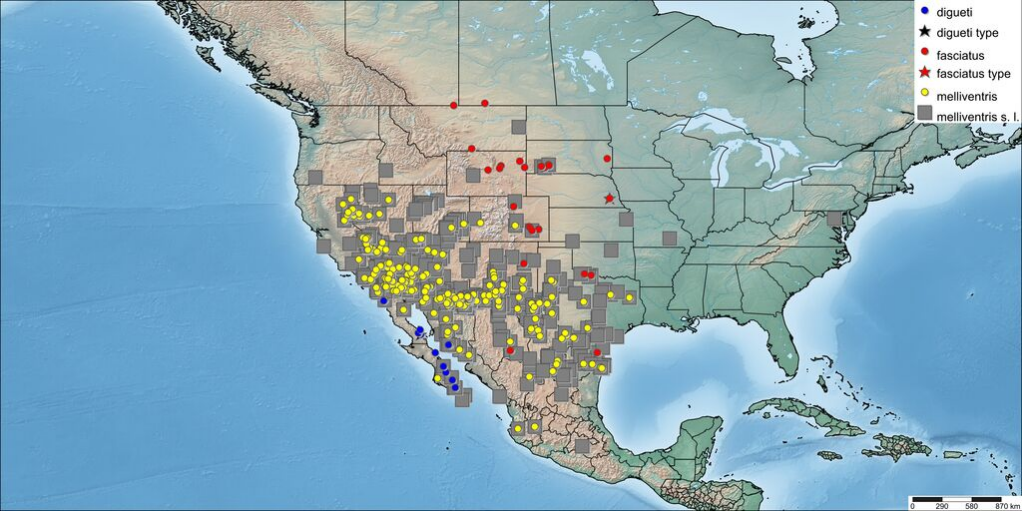
Distribution of confirmed specimens of *Agapostemonfasciatus* Crawford, 1901 (red dots - data provided above) and *A.melliventris* Cresson, 1874 (yellow dots, including type material of *A.digueti* Cockerell, 1924 shown in blue dots) and material identified as *A.melliventris* s. l., but not examined from GBIF.org (2023) and as recorded in literature (underlying grey boxes).

In addition to these morphological differences in both sexes, the distribution of *A.fasciatus* is typically more northern across the Great Plains, extending into southern Canada (Alberta, Saskatchewan), with only a few records occurring in the south-western United States (Fig. 7).

## Analysis

In addition to the distinctive morphological characters of both sexes of *A.fasciatus* described above, which are consistent across all material examined, it also differs from *A.melliventris* genetically, based on results from the Barcode Gap Analysis tool in BOLD. *Agapostemonfasciatus* is presently assigned to Barcode Index Number (BIN, after Ratnasingham and Hebert (2013)) BOLD:ACU3900 and differs by 7.99% sequence divergence from other "*A.melliventris*" in BOLD (currently represented by at least three BINs BOLD:AAJ1185, BOLD:ABY2743 and BOLD:AAN8220, which will be the subject of a subsequent paper on the remaining members of species complex not found in Canada). Amongst these four BINs, the mean distance in sequence divergence averages 8.82%, suggesting multiple additional taxa.

## Discussion

Crawford (1901) mentioned that 18 female and 37 male species formed the type series (i.e. syntypes), though none of these was specifically designated as a lectotype within any subsequent publication by that author. However, Sandhouse (1936) indicated that Crawford selected a lectotype female and lectoallotype male from his syntype series (these also examined by Cockerell (1937) and Roberts (1972)) from material at the USNM (see above).

Roberts (1972) indicated that he also examined the type material of *A.fasciatus* and *A.digueti* and believed they were conspecific with *A.melliventris*, based on variable metasomal colour of the females alone and pointed out that, until he synonymised *A.fasciatus* with *A.melliventris*, it had been considered by some authors to be of subspecific rank (e.g. Sandhouse (1936)), though others continued to consider it a valid species (e.g. Cockerell (1937), Fischer (1950)). His (Roberts 1972) argument for the synonymy was also based on his belief that *A.fasciatus* was sympatric with *A.melliventris* in the south-western part of the range (namely Arizona) where intermediate forms were "too common" (cf. discussion of variation). However, there is no reason why distinct species cannot have overlapping ranges and it is likely that he made this comment based on female metasomal colour alone (which varies even with *A.melliventris* s. str.); he may not have examined or noted the other characters indicated above that seem to be consistent within each taxa (i.e. scape maculations in females and leg colouring in males). When these are considered, a differing distribution of the two taxa is observed, with only a small area of sympatry (Fig. 7). Thus, some of the material identifed as *A.melliventris* from the Great Plains reported recently by Portman et al. (2022) may actually be *A.fasciatus*.

Roberts (1972) also commented on the nomenclature of *A.fasciatus*. Vachal (1903) considered AgapostemonasubgenusofHalictus Latreille, 1804, making Crawford’s species a junior secondary homonym of *Halictusfasciatus* Nylander, 1848 (=*Halictustumulorum* (Linnaeus, 1758)), so Vachal (1903) proposed the new name, Halictus (Agapostemon) plurifasciatus Vachal 1903. The same also occurred for *A.coloradensis* Crawford, 1901, which Vachal (1903) renamed H. (A.) coloradinus Vachal, 1903. These specific epithets were used by Sandhouse (1936), though Cockerell (1937) and Michener (1951) believed Crawford's epithet should be used for *A.fasciatus* and *A.coloradensis*; a petition from T.D.A Cockerell to the ICZN regarding Crawford's *A.coloradensis* caused much debate after his death on the larger issues created by these *Agapostemon* species (see Hemming (1950)). However, Roberts (1972) indicated, based on the International Code of Zoological Nomenclature (citing ICZN (1964)), "secondary homonyms rejected before 1961 cannot be revived," specifically citing the example of *A.coloradinus* which has been used as a valid species name since Vachal (1903) (e.g. Sandhouse (1936), Roberts (1972), Hurd (1979), Moure and Hurd (1987)). Roberts (1972) also suggested that, if Crawford’s *A.fasciatus* were to be recognised as a valid taxon (as is done so here), then *A.plurifasciatus* should be used, but this is not the case as (from ICZN (1999): 59.3. Secondary homonyms replaced before 1961 but no longer considered congeneric): “A junior secondary homonym replaced before 1961 is permanently invalid unless the substitute name is not in use [applicable to *A.plurifasciatus* since it was synonymised with *A.melliventris* by Sandhouse (1936) and Roberts (1972)] and the relevant taxa are no longer considered congeneric, in which case the junior homonym is not to be rejected on grounds of that replacement”. As such, *A.fasciatus* is resurrected from synonymy with *A.melliventris* and Crawford’s original species epithet is considered valid. By contrast, as *A.coloradinus* has remained in use in a number of taxonomic (e.g. Sandhouse (1936), Roberts (1972), Hurd (1979), Moure and Hurd (1987), Janjic and Packer (2003), Scott et al. (2011)) and ecological works (e.g. Lavigne (1976), Berger et al. (1985), Osborn et al. (1988)) since Vachal (1903), it is the appropriate name for that taxon (see ICZN (1999)).

Specimens from at least three additinal BINs are tentatively identified in BOLD as *A.melliventris*, mostly all matching the general discription of *A.melliventris*, but suggesting that there are possible additional species in this species group. Members of BOLD:AAJ1185 (seven male specimens) are from one location on the Texas/Mexico border and members assigned to BOLD:ABY2743 are from Arizona (two female, three male specimens). Most interesting are the four specimens of BOLD:AAN8220, also from Arizona, though with the single female having both the clypeus apically and scape maculated, though with a metasoma that is similar to *A.fasciatus* (based on images available on BOLD). These materials will be covered in a subsequent work on the *A.melliventris* species complex, which is currently in progress.

## Supplementary Material

XML Treatment for Agapostemon (Agapostemon) fasciatus

## Figures and Tables

**Figure 2a.**
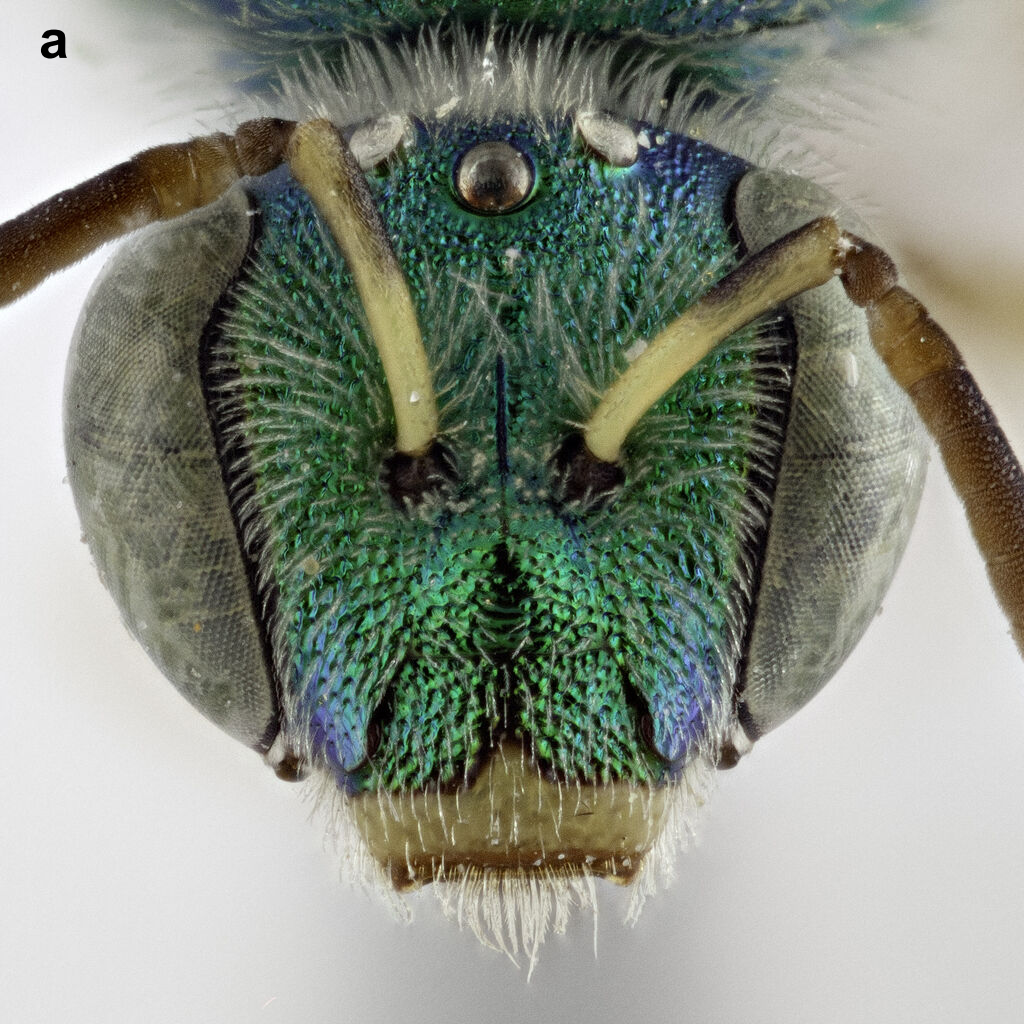
*Agapostemonmelliventris* Cresson, 1874 with pale maculations on clypeus apically, and on scape;

**Figure 2b.**
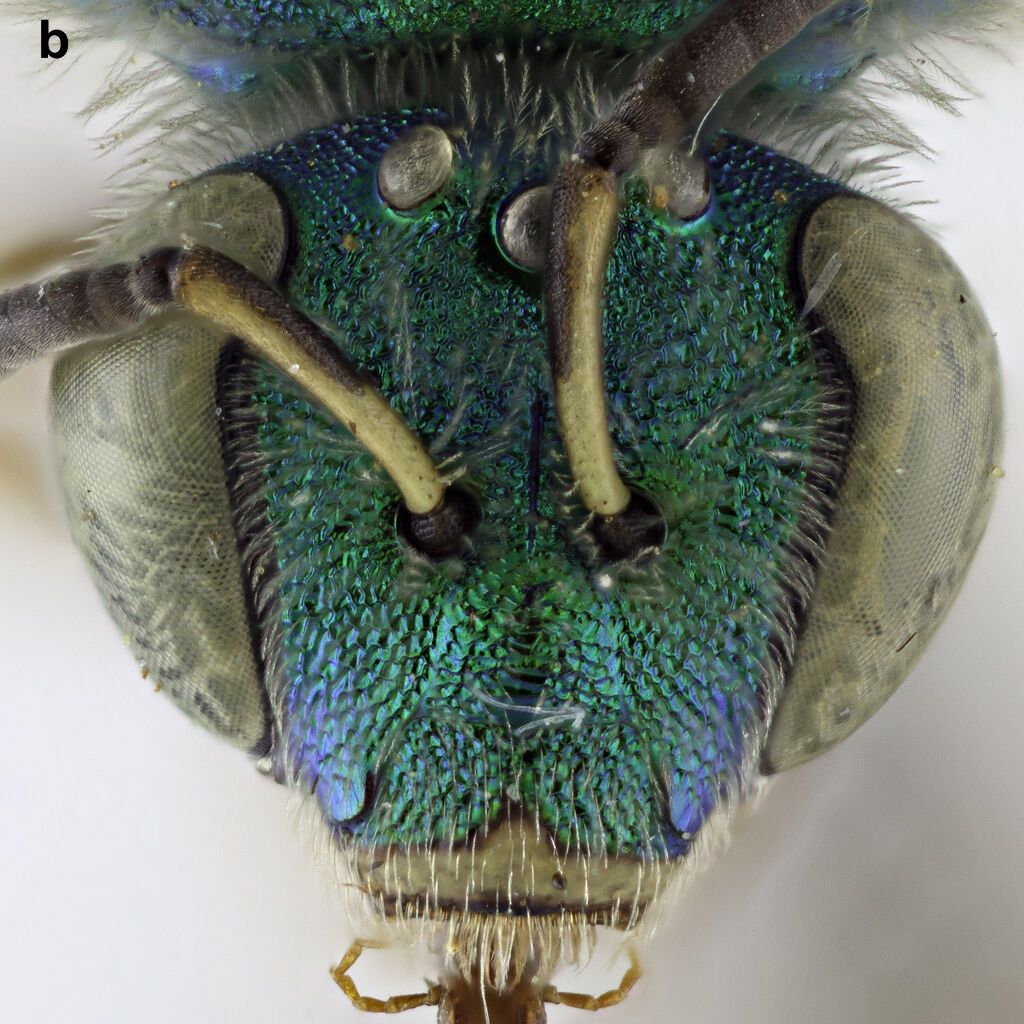
*Agapostemondigueti* Cockerell, 1924 (= *A.melliventris* Cresson, 1874) with pale maculations on clypeus apically and on scape. Holotype;

**Figure 2c.**
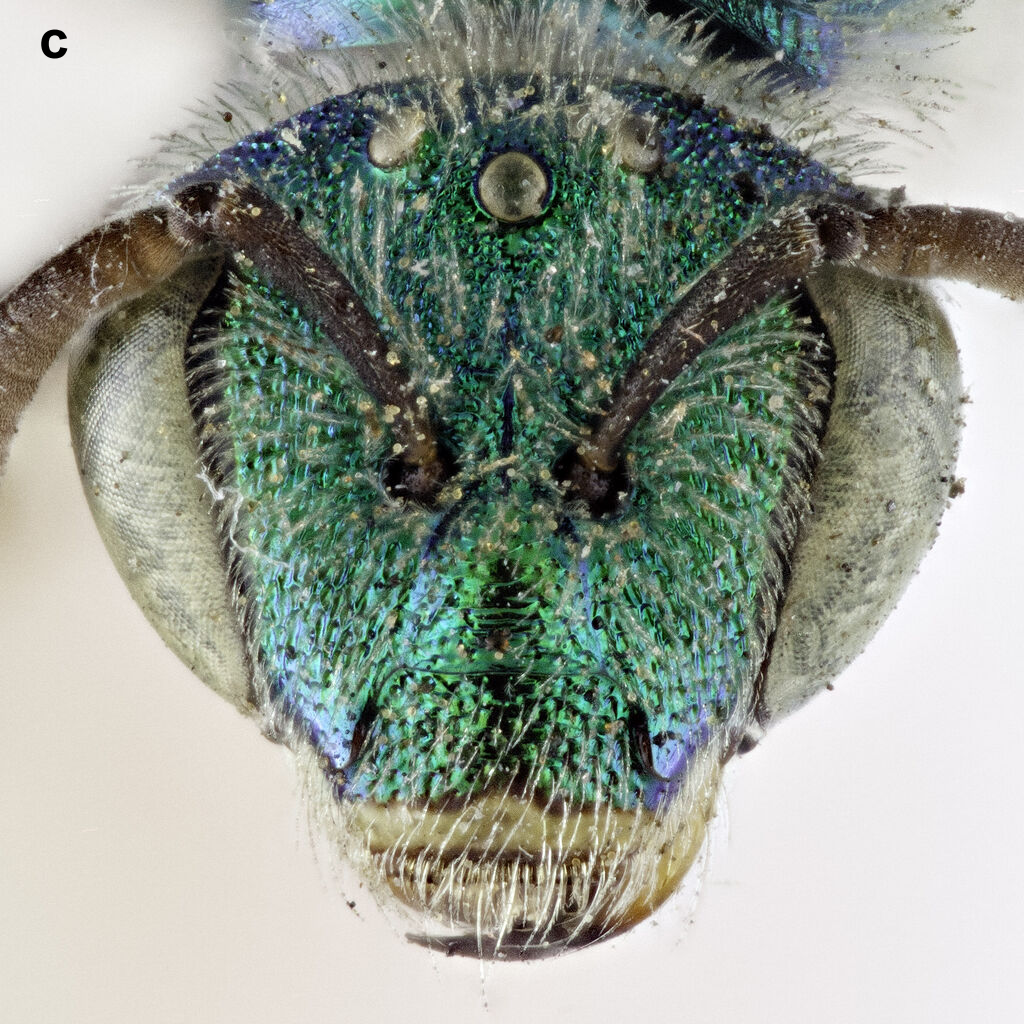
*Agapostemonfasciatus* Crawford, 1901 with pale maculations on clypeus apically, but not on scape;

**Figure 2d.**
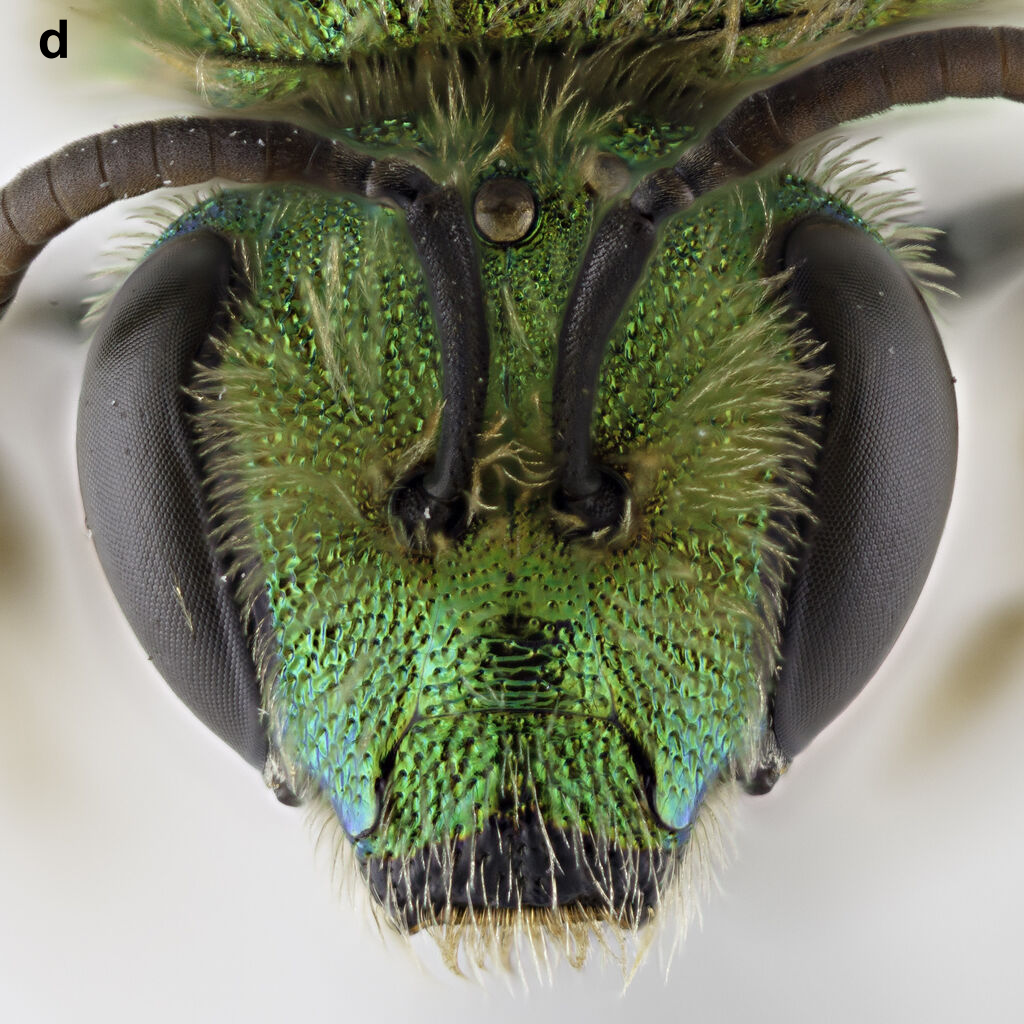
*Agapostemonvirescens* (Fabricius, 1775) with no maculation on clypeus apically or on scape.

**Figure 3a.**
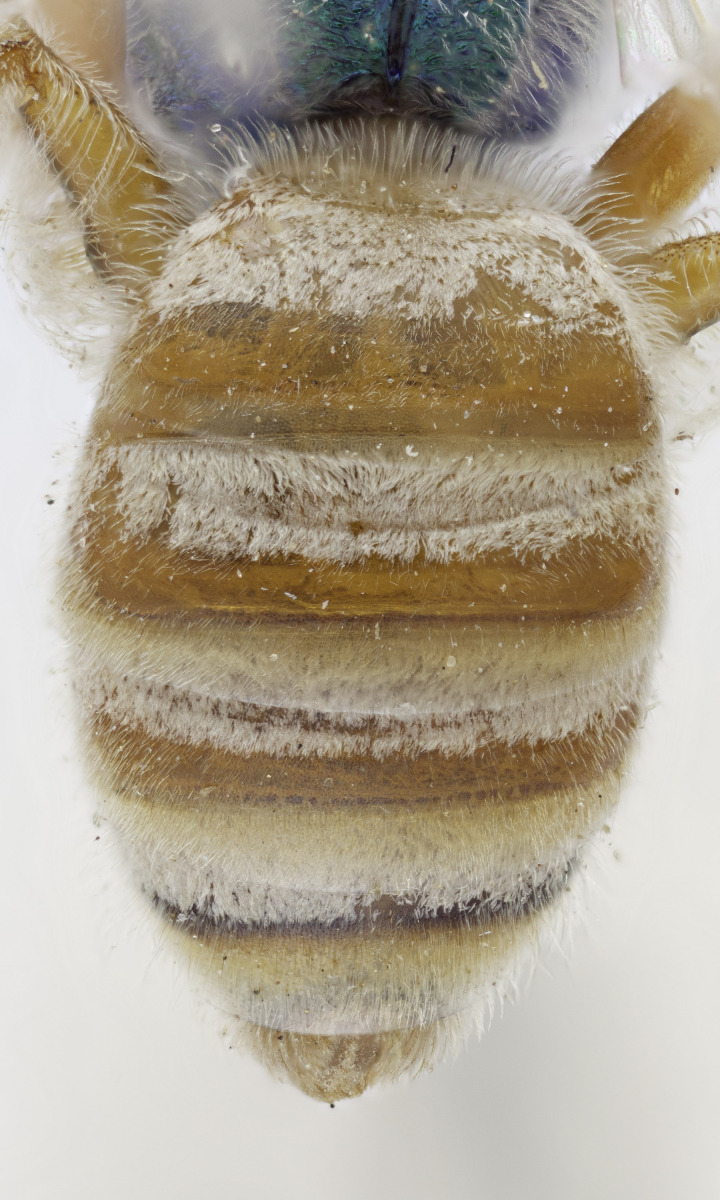
*Agapostemonmelliventris* Cresson, 1874; typical pattern;

**Figure 3b.**
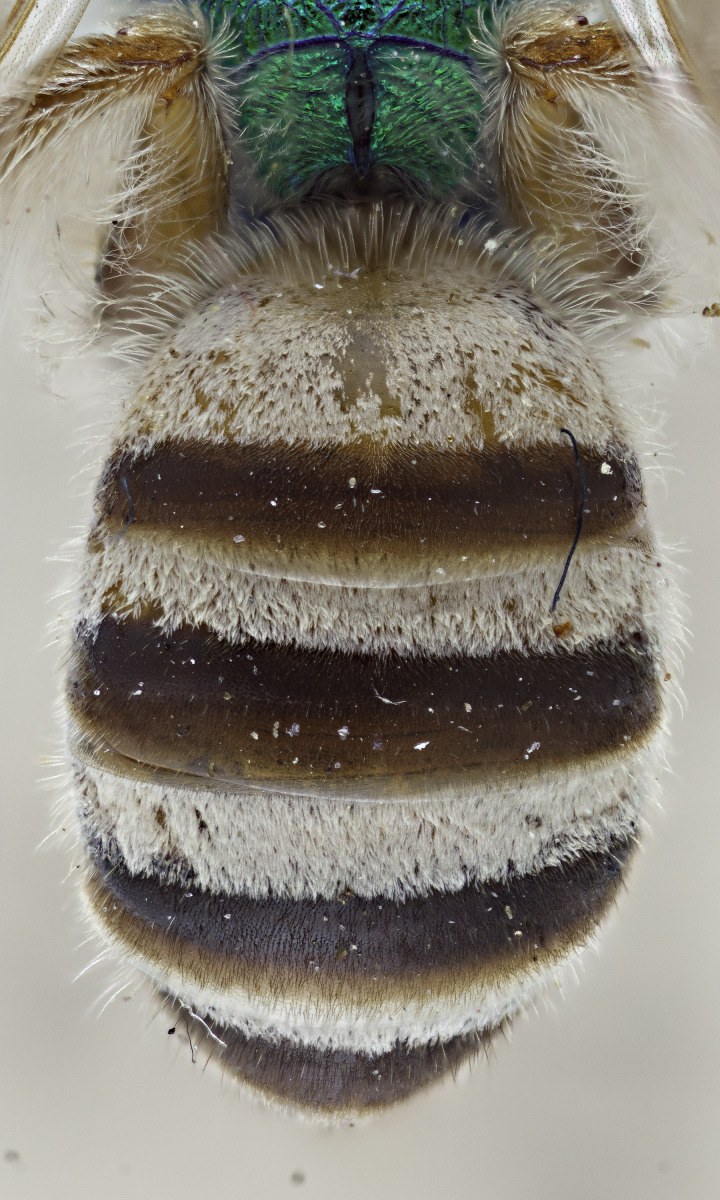
*Agapostemondigueti* Cockerell, 1924; paratype.

**Figure 4a.**
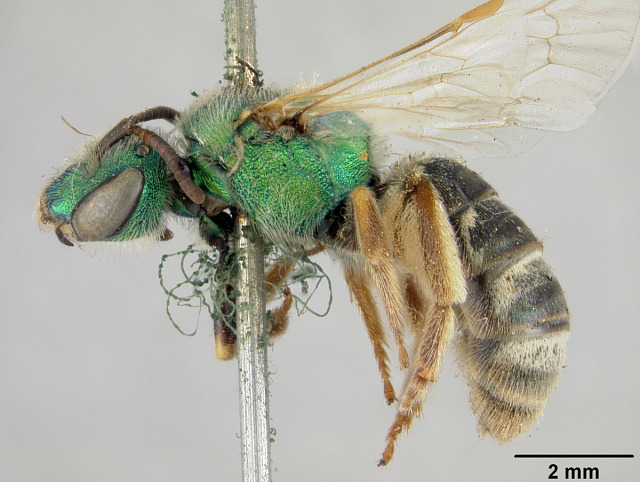
Lateral view;

**Figure 4b.**
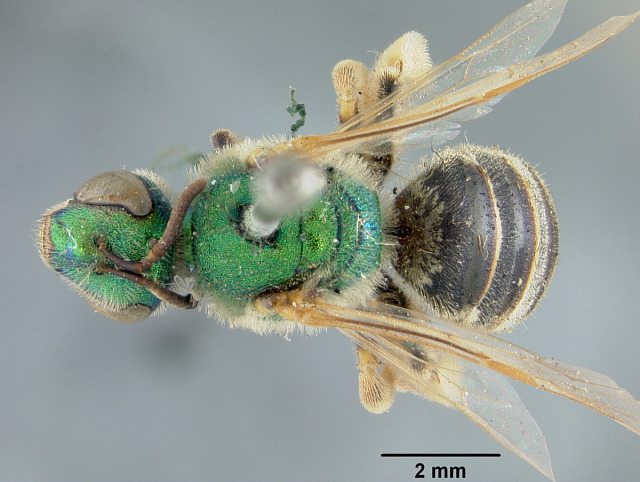
Dorsal view;

**Figure 4c.**
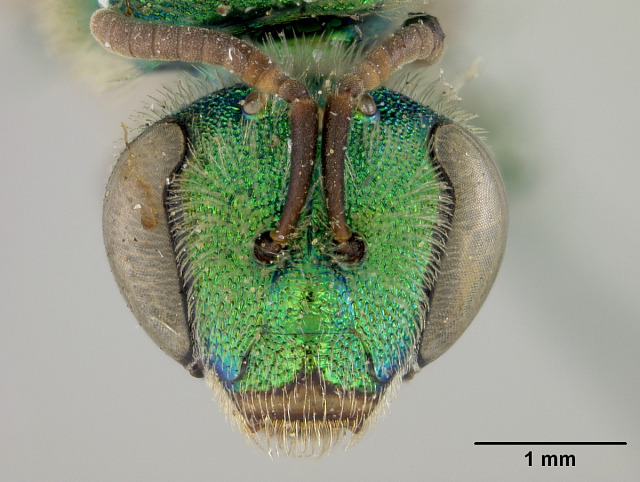
Face, showing clypeus with pale apical maculation, but unmaculated scape;

**Figure 4d.**
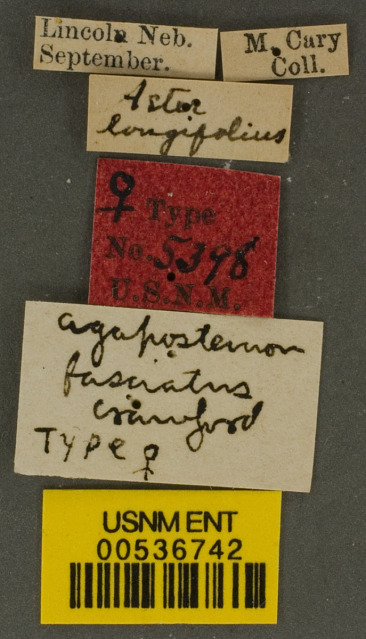
Associated labels.

**Figure 5a.**
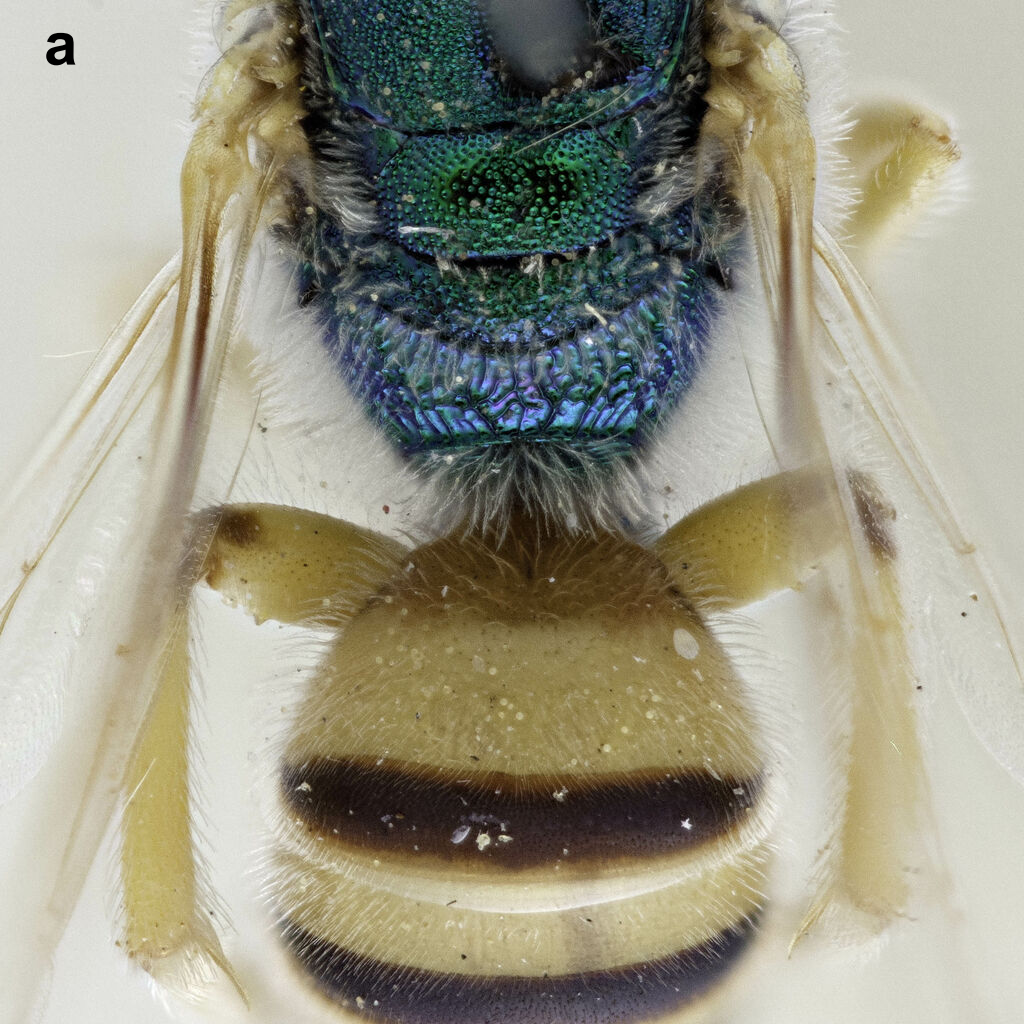
*Agapostemonfasciatus* Crawford, 1901;

**Figure 5b.**
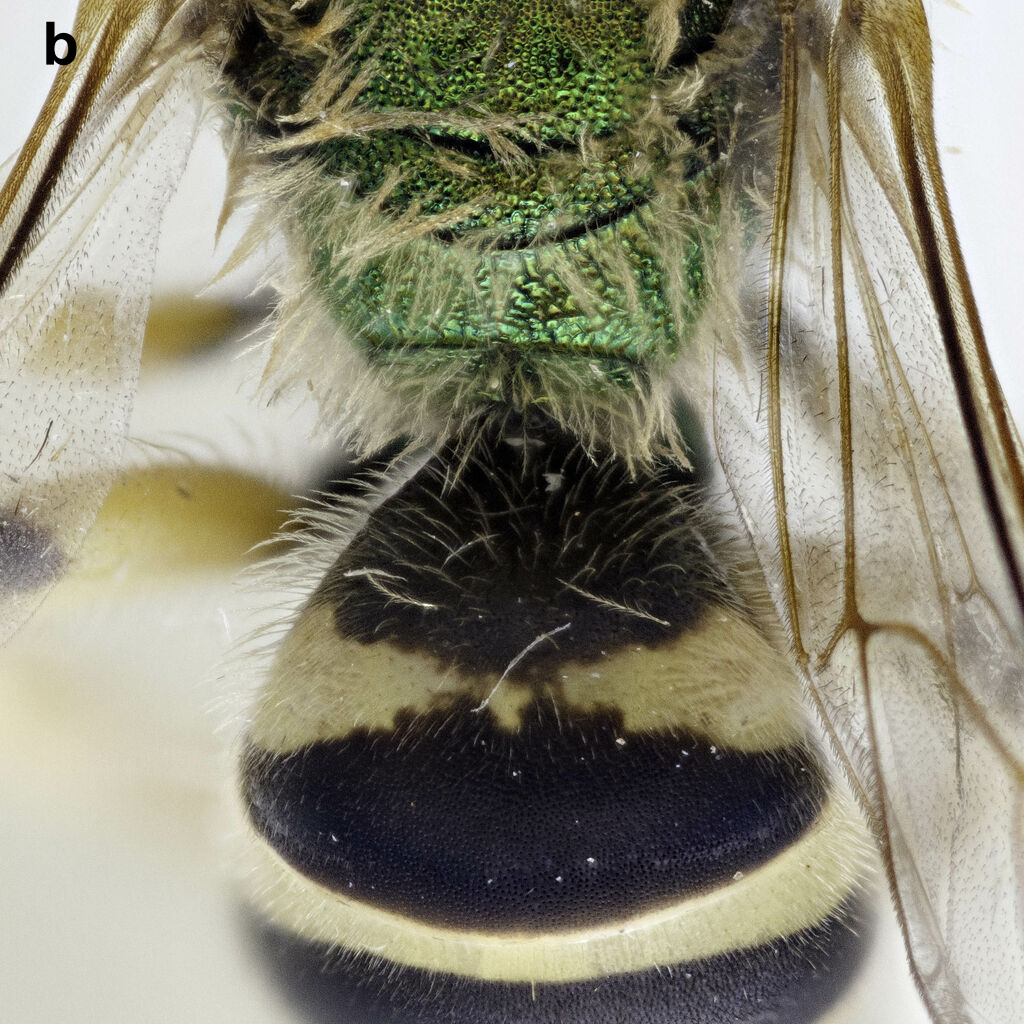
*Agapostemonvirescens* (Fabricius, 1775).

**Figure 6a.**
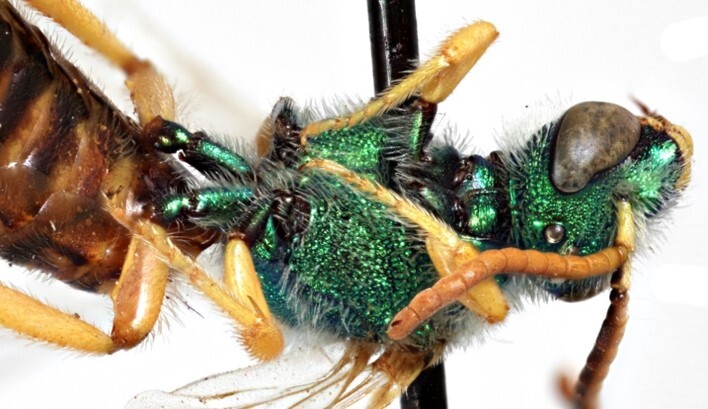
*Agapostemonfasciatus* Crawford, 1901 (paralectotype), showing the metallic green coxae and trochanters of each leg;

**Figure 6b.**
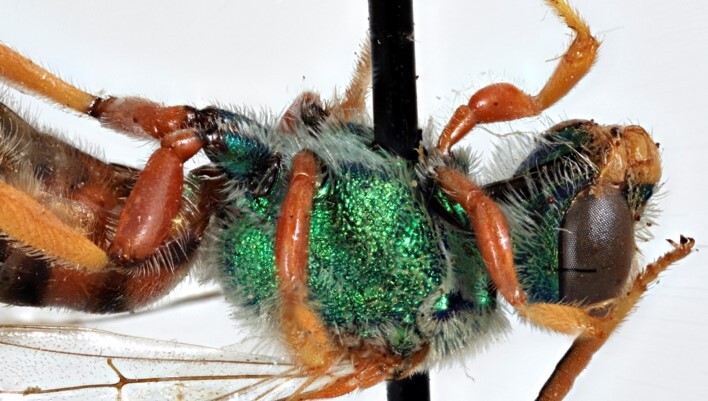
*Agapostemonmelliventris* Cresson, 1874, showing the metallic green coxae and non-metallic trochanters of each leg; this pattern is also consistent with *A.digueti* Cockerell, 1924.
